# Characterization of the Wheat Heat Shock Factor TaHsfA2e-5D Conferring Heat and Drought Tolerance in *Arabidopsis*

**DOI:** 10.3390/ijms23052784

**Published:** 2022-03-03

**Authors:** Huihui Bi, Jingnan Miao, Jinqiu He, Qifan Chen, Jiajun Qian, Huanhuan Li, Yan Xu, Dan Ma, Yue Zhao, Xuejun Tian, Wenxuan Liu

**Affiliations:** 1 National Key Laboratory of Wheat and Maize Crop Science, Henan Agricultural University, Zhengzhou 450002, China; huihui.bi@henau.edu.cn (H.B.); mjn1006m@163.com (J.M.); hejinqiu1015@163.com (J.H.); chenqifan02@163.com (Q.C.); qanjiajun@163.com (J.Q.); lihuanhuanhappy@henau.edu.cn (H.L.); liuwenxuan@henau.edu.cn (W.L.); 2 College of Bioengineering, Jingchu University of Technology, Jingmen 448000, China; xuyan2018@jcut.edu.cn (Y.X.); 200308006@jcut.edu.cn (D.M.)

**Keywords:** wheat, Hsf, environmental stresses, transgenic, *Arabidopsis*, expression

## Abstract

Environmental stresses, especially heat and drought, severely limit plant growth and negatively affect wheat yield and quality worldwide. Heat shock factors (Hsfs) play a central role in regulating plant responses to various stresses. In this study, the wheat heat shock factor gene *TaHsfA2e-5D* on chromosome 5D was isolated and functionally characterized, with the goal of investigating its role in responses to heat and drought stresses. Gene expression profiling showed that *TaHsfA2e-5D* was expressed constitutively in various wheat tissues, most highly in roots at the reproductive stage. The expression of *TaHsfA2e-5D* was highly up-regulated in wheat seedlings by heat, cold, drought, high salinity, and multiple phytohormones. The TaHsfA2e-5D protein was localized in the nucleus and showed a transcriptional activation activity. Ectopic expression of the *TaHsfA2e-5D* in yeast exhibited improved thermotolerance. Overexpression of the *TaHsfA2e-5D* in *Arabidopsis* results in enhanced tolerance to heat and drought stresses. Furthermore, RT-qPCR analyses revealed that *TaHsfA2e-5D* functions through increasing the expression of Hsp genes and other stress-related genes, including *APX2* and *GolS1*. Collectively, these results suggest that *TaHsfA2e-5D* functions as a positive regulator of plants’ responses to heat and drought stresses, which may be of great significance for understanding and improving environmental stress tolerance in crops.

## 1. Introduction

Wheat (*Triticum aestivum* L.) is one of the most important staple food crops in the world. Environmental stresses, such as high temperatures and drought, adversely affect wheat viability and yield globally, especially during pollination and grain-filling stages. Plants have developed various morphological, physiological, biochemical and molecular alterations, and adaptation strategies to acquire stress tolerance [1,2]. One of the effective strategies is to transcriptionally regulate the expression of stress-related genes, among which transcription factors play a crucial role by activating and/or inhibiting specific genes [3]. Heat shock factors (Hsfs) are one of the prime transcription factors identified in plants in response to heat stress, which are responsible for activation of heat-responsive genes (for example, Hsp genes) by recognizing conserved heat shock elements (HSEs) in their promoters [4,5]. Hsps rapidly accumulate in response to heat stress, protecting plants against heat stress by re-establishing normal protein conformation, thereby maintaining cellular homeostasis [6]. Hsfs are structurally and functionally conserved throughout eukaryotes, and they consist of a common core structure comprising a DNA-binding domain (DBD) and an oligomerization domain (OD or HR-A/B) [7]. The N-terminal DBD is a typical structure in Hsfs that specifically recognizes HSEs [8]. The adjacent HR-A/B is responsible for Hsf trimerization during transcriptional activation. Hsfs can be divided into three classes (HsfA, HsfB and HsfC) based on the different linkers between HR-A and HR-B [9]. In addition, the nuclear localization signal (NLS), nuclear export signal (NES) and C-terminal transcriptional activation domains (CTAD or AHA) are found in some Hsfs. The conserved DBDs and variable AHAs imply evolutionarily functional conservation and divergence [10].

Hsfs are widespread in plants and play important roles in various plant processes including the regulation of growth and development, signaling networks, responses to abiotic stresses, etc. Several wheat Hsfs have been functionally characterized, mainly focusing on their roles in abiotic stress responses. For instance, overexpression of the seed-preferential *TaHsfA2d* provided increased heat and salt tolerance in *Arabidopsis* [11]; additionally, it (renamed as *TaHsfA6b*) improved thermotolerance in transgenic barley [12]. *Arabidopsis* overexpressing *TaHsfA2-1* or *TaHsfA2-10* displayed enhanced thermotolerance by up-regulating the expression of Hsp genes [13,14]. *TaHsfA4a* conferred cadmium tolerance by increasing the expression levels of metallothionein genes in rice [15]. *TaHsfA6f* overexpression resulted in improved tolerance to heat, drought and salt stresses in *Arabidopsis* [16], and as a transcription activator directly regulating *TaHSP*, *TaGAAP* and *TaRof1* genes, *TaHsfA6f* exerted a positive effect on thermotolerance in wheat [17]. *TaHsfA6e* modulates heat and drought tolerance in wheat by regulating the expression of Hsp genes, such as *Hsp17*, *Hsp70* and *Hsp90* [18]. Overexpression of *TaHsf3* (belonging to class HsfB) enhanced tolerance to extreme temperatures in *Arabidopsis* by activating Hsp genes, such as *Hsp70* [19]. *TaHsfC2a* can improve thermotolerance through an ABA-mediated regulatory network in wheat [20]. Screening of wheat genome data by different researchers has revealed that 53–82 Hsfs are present in wheat, and the expression of these Hsfs varies greatly across different wheat tissues and in response to various abiotic stresses [21,22,23,24]. This has led us to hypothesize that other uncharacterized wheat Hsfs should also function in abiotic stress responses.

In this study, we identified a Hsf gene on chromosome 5D from the heat-/drought-resistant wheat cultivar TAM107 and designated it as *TaHsfA2e-5D*. To unravel the biological function of *TaHsfA2e-5D*, we investigated its expression patterns, subcellular localization, transactivation activity, and stress tolerance in transgenic yeast and *Arabidopsis*. The findings provide evidence that *TaHsfA2e-5D* plays a significant role in plant responses to heat and drought stresses, and that *TaHsfA2e-5D* represents a valuable candidate gene for engineering heat and drought tolerant plants in future.

## 2. Results

### 2.1. TaHsfA2e-5D Expression Is Induced by Various Abiotic Stresses and Phytohormones

**Figure 1:**
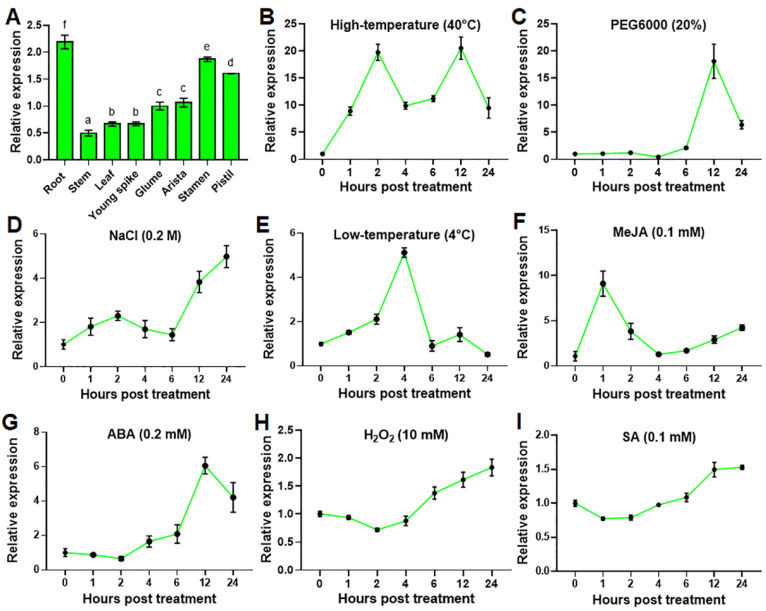
Expression analyses of *TaHsfA2e-5D*. (**A**) Expression levels of *TaHsfA2e-5D* in different tissues of TAM107 wheat at the booting stage. Different lowercase letters above the bars denote significant differences at the *p* < 0.05 level. (**B**–**I**) Expression patterns of *TaHsfA2e-5D* during abiotic stresses and phytohormones treatment. Total RNA was extracted from 10-day-old wheat seedlings treated with 40 °C high-temperature (**B**), 20% PEG6000 (**C**), 0.2 M NaCl (**D**), 4 °C low-temperature (**E**), 0.1 mM MeJA (**F**), 0.2 mM ABA (**G**), 10 mM H_2_O_2_ (**H**), and 0.1 mM SA (**I**), for the indicated time points. The wheat *β-actin* gene was used as an internal control. All data represent means ± standard deviation (SD) of three replicates.

By digging into published transcriptome sequencing data of wheat (TAM107) seedlings under normal growth conditions and heat stress for 6 h [25], we identified a heat shock factor gene, *TaHsfA2e-5D*, which was highly up-regulated by heat stress and thus was selected for further functional characterization. Firstly, we investigated the expression profiles of *TaHsfA2e-5D* in different wheat tissues. As shown in Figure 1A, *TaHsfA2e-5D* was expressed in all parts of wheat plants examined; the highest expression was noted in the root, stamen and pistil, followed by that in the glume and arista, whereas the lowest expression was found in the leaf, young spike, and stem under normal growth conditions. Next, we explored the expression patterns of *TaHsfA2e-5D* in response to various abiotic stresses and phytohormones. Under a high-temperature, low-temperature and methyl jasmonate (MeJA) treatments, the expression levels of *TaHsfA2e-5D* were up-regulated rapidly and peaked at 2, 4 and 1 h, respectively (Figure 1B,E,F). *TaHsfA2e-5D* expression was induced by PEG6000 or abscisic acid (ABA) at 6 h post treatment, peaked at 12 h, and then decreased sharply (Figure 1C,G). Following NaCl stress, the *TaHsfA2e-5D* transcript increased gradually from 6 h (Figure 1D). For H_2_O_2_ and salicylic acid (SA) treatments, the responses of *TaHsfA2e-5D* were relatively weak, and its mRNA levels slightly increased after 6 h of treatment (Figure 1H,I). These results implied that *TaHsfA2e-5D* appears to be involved in different signal transduction pathways and may play an important role in various abiotic stress responses in plants.

### 2.2. Molecular Characterization of TaHsfA2e-5D

**Figure 2:**
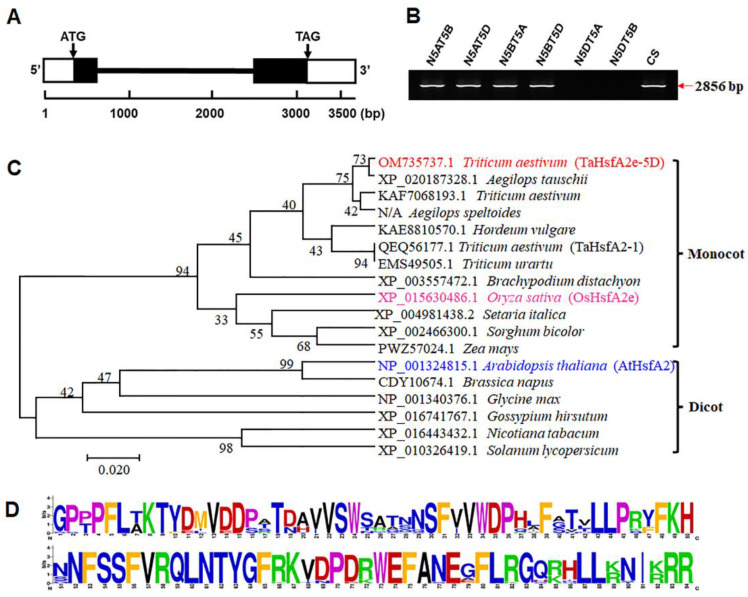
Molecular characterization of *TaHsfA2e-5D*. (**A**) Gene structure of *TaHsfA2e-5D*. White boxes, black boxes, and black line represent untranslated regions, exons, and intron, respectively. (**B**) Chromosomal location of TaHsfA2e-5D in CS NT lines. N5AT5B: nullisomic 5A-tetrasomic 5B; N5AT5D: nullisomic 5A-tetrasomic 5D; N5BT5A: nullisomic 5B-tetrasomic 5A; N5BT5D: nullisomic 5B-tetrasomic 5D; N5DT5A: nullisomic 5D-tetrasomic 5A; N5DT5B: nullisomic 5D-tetrasomic 5B; CS: Chinese Spring. (**C**,**D**) Phylogenetic relationships (**C**) and sequences logo (**D**) of the DBD domain of TaHsfA2e-5D and its homologous proteins from other species.

The genomic and coding sequence alignment showed that *TaHsfA2e-5D* (GenBank accession number OM735737) included two exons and contained a 1047-bp open reading frame encoding 348 amino acid residues (Figure 2A and Figure S1). The gene-specific amplicons of *TaHsfA2e-5D* were observed in four Chinese Spring (CS) nulli-tetrasomic (NT) lines of chromosome 5, but not in lines N5DT5A (nullisomic 5D-tetrasomic 5A) and N5DT5B (nullisomic 5D-tetrasomic 5B), indicating that *TaHsfA2e-5D* was located on the chromosome 5D (Figure 2B). Phylogenetic analyses indicated that TaHsfA2e-5D and its homologs diverged distinctly between monocot and dicot species (Figure 2C). TaHsfA2e-5D shared the highest similarity with the *Aegilops tauschii* (the D genome donor species of hexaploid wheat) protein XP_020187328.1, and it was homologous to rice OsHsfA2e (XP_015630486.1) (Figure 2C). In addition, it was found that the TaHsfA2e-5D protein owned a typical DBD domain at the N-terminal, which exhibited significant sequence conservation within Hsf proteins from various species (Figure 2D and Figure S1).

### 2.3. Promoter Isolation and Cis-Acting Regulatory Elements Analysis of TaHsfA2e-5D

**Figure 3:**
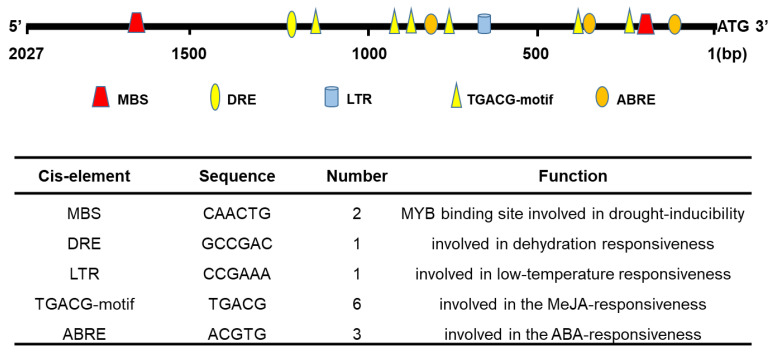
Putative cis-acting elements in the 5′-flanking region of *TaHsfA2e-5D*. A region of 2027 bp upstream of the start codon of *TaHsfA2e-5D* was isolated as its 5′-flanking region and used for the prediction of cis-acting elements. MBS: MYB binding site element; DRE: dehydration-responsive element; LTR: low-temperature-responsive element; ABRE: ABA-responsive element.

To understand the underlying mechanism responsible for the regulation of *TaHsfA2e-5D* expression, we isolated a 2027 bp DNA sequence upstream of the start codon of *TaHsfA2e-5D* using a PCR strategy. We searched for putative cis-acting regulatory elements in this sequence using PlantCARE (Figure 3). A number of stress-related elements were identified, including drought-responsive element (MBS), dehydration-responsive element (DRE), low-temperature-responsive element (LTR), MeJA-responsive element (CGTCA/TGACG-motif) and ABA-responsive element (ABRE).

### 2.4. TaHsfA2e-5D Is a Nucleus-localized Transcriptional Activator

**Figure 4:**
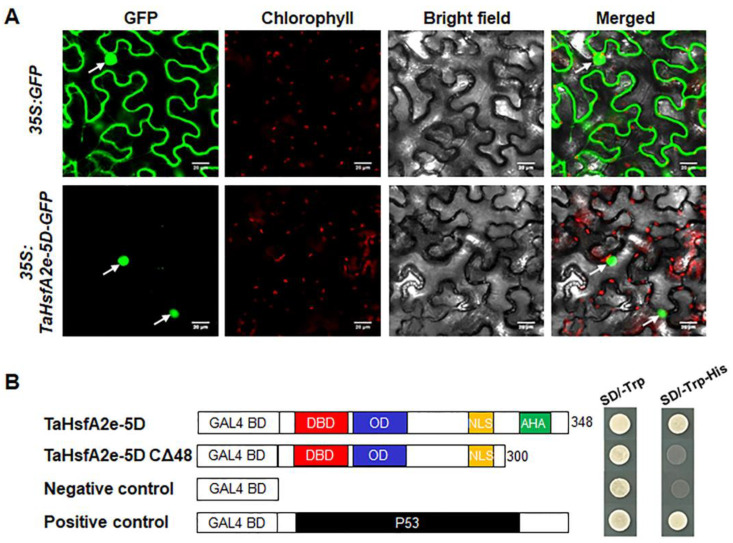
Subcellular localization and transcriptional activity analyses of TaHsfA2e-5D. (**A**) Subcellular localization of TaHsfA2e-5D in tobacco leaf epidermal cells. White arrows indicate nuclei. Bars = 20 μm. (**B**) Transcriptional activation of TaHsfA2e-5D in yeast. Schematic representation of the full-length TaHsfA2e-5D and truncated protein constructs in the pGBKT7 vector. Fusion proteins of the GAL4 DNA-binding domain (BD) and full-length TaHsfA2e-5D or truncated TaHsfA2e-5D with the deletion of 48 C-terminal amino acid residues (CΔ48) were expressed in the yeast strain AH109. The pGBKT7 and pGBKT7-P53 vectors were used as negative and positive controls, respectively.

Domain analyses revealed that TaHsfA2e-5D possesses NLS and AHA motifs similar to other known HsfAs (Figure S1), indicating that TaHsfA2e-5D is likely to be a nucleus-localized transcriptional activator. In the assessment of subcellular localization of TaHsfA2e-5D through transient transformation in tobacco leaf epidermal cells, the green fluorescence of cells with GFP was distributed throughout the cell, whereas it was only observed in the nucleus of cells with TaHsfA2e-5D-GFP, verifying that TaHsfA2e-5D is a nucleus-localized protein (Figure 4A). The transactivation activity of TaHsfA2e-5D was evaluated with the GAL4 yeast system. As shown in Figure 4B, all transformants grew well on SD/-Trp medium. However, only the yeast harboring pGBKT7-TaHsfA2e-5D and pGBKT7-P53 constructs, but not the yeast carrying pGBKT7-TaHsfA2e-5D CΔ48 and pGBKT7 vectors, grew well on SD/-Trp-His medium, confirming that TaHsfA2e-5D functions as a transcriptional activator and the predicted AHA motif is required for its transactivation activity.

### 2.5. Ectopic Expression of TaHsfA2e-5D Improves Thermotolerance in Yeast

**Figure 5:**
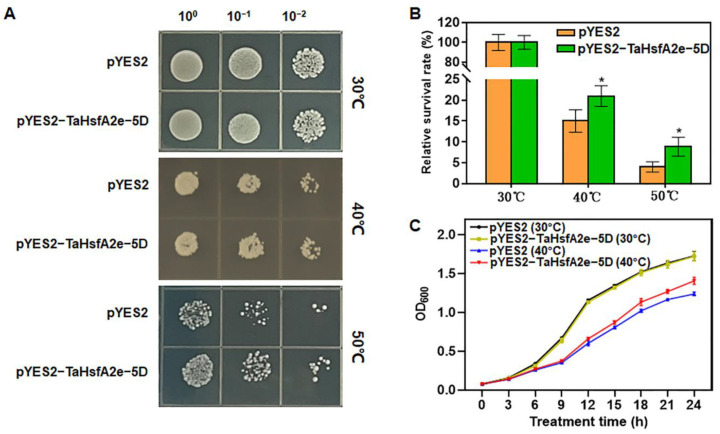
Thermotolerance assays of transgenic yeast with pYES2 vector alone and pYES2-TaHsfA2e-5D recombinant vector. (**A**) Growth status of transgenic yeast before (30 °C) and after heat stress (40 °C or 50 °C, 1 h). 10^0^, 10^−1^ and 10^−2^ represent the 10-fold serial gradient dilutions. (**B**) Relative survival rates of yeast harboring pYES2 or pYES2-TaHsfA2e-5D treated with 40 °C or 50 °C for 1 h. (**C**) Growth curves of transgenic yeast under normal (30 °C) and heat stress (40 °C) conditions. Values are means ± SD of triplicate experiments. * indicates *p* < 0.05 (Student’s *t*-test).

The aforementioned results indicated that the expression of *TaHsfA2e-5D* was remarkably induced by heat stress (Figure 1B). To confirm the function of *TaHsfA2e-5D* in heat stress responses, we used ectopic expression in yeast to study its effect on thermotolerance. Under normal growth conditions (30 °C), no obvious difference was observed in the growth status between the yeast transformed with pYES2 and that transformed with pYES2-TaHsfA2e-5D, and the growth curves of the two kinds of transformants almost overlapped (Figure 5A,C). However, after heat treatments (40 °C or 50 °C), the growth of both transformants was inhibited, but the pYES2-TaHsfA2e-5D transgenic yeast grew better and had higher survival rates compared with the pYES2 transgenic yeast (Figure 5A,B). Moreover, the OD600 values of pYES2-TaHsfA2e-5D transgenic yeast were obviously higher than those of pYES2 transgenic yeast after 9 h of treatment (Figure 5C). These results demonstrated that ectopic expression of *TaHsfA2e-5D* improves thermotolerance in yeast.

### 2.6. Overexpression of TaHsfA2e-5D Confers Heat and Drought Tolerance in Arabidopsis

**Figure 6:**
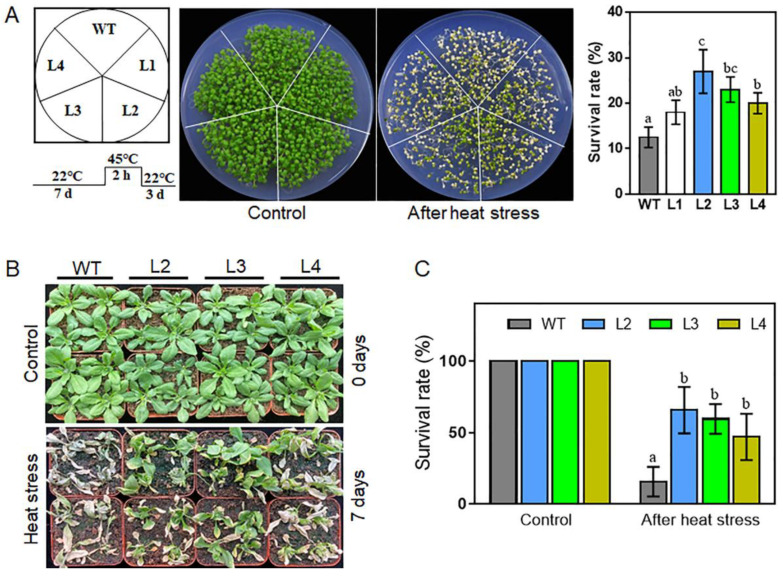
Thermotolerance assays of WT and *TaHsfA2e-5D* overexpressing transgenic *Arabidopsis* lines. (**A**) Morphology and survival rates of WT and transgenic lines (L1, L2, L3 and L4) grown on MS agar plates after heat treatment. Seven-day-old seedlings were treated at 45 °C for 2 h and recovered at 22 °C for 3 days. (**B**,**C**) Comparison of thermotolerance among WT and transgenic lines (L2, L3, and L4) in soil. Phenotype (**B**) and survival rates (**C**) of WT and transgenic lines after heat stress at 40/32 °C (day/night) for 7 days. For each experiment, at least 20 plants per line were used; values are means ± SD from three independent measurements. Different lowercase letters above the bars denote significant differences at the *p* < 0.05 level.

To further determine the usefulness of *TaHsfA2e-5D* in molecular breeding for plant thermotolerance, four single copy *TaHsfA2e-5D* overexpressing lines (L1, L2, L3 and L4) displaying different transcript levels (Figure S2) were selected to examine the role of *TaHsfA2e-5D* in thermotolerance in *Arabidopsis*. As shown in Figure 6A,B, under normal growth conditions, no visible phenotype variation was observed between WT and transgenic lines. However, under heat stress conditions, the *TaHsfA2e-5D* overexpressing lines were more resistant to heat stress than WT in terms of seedling survival rates (Figure 6). These results proved that overexpression of *TaHsfA2e-5D* confers thermotolerance in *Arabidopsis*.

**Figure 7:**
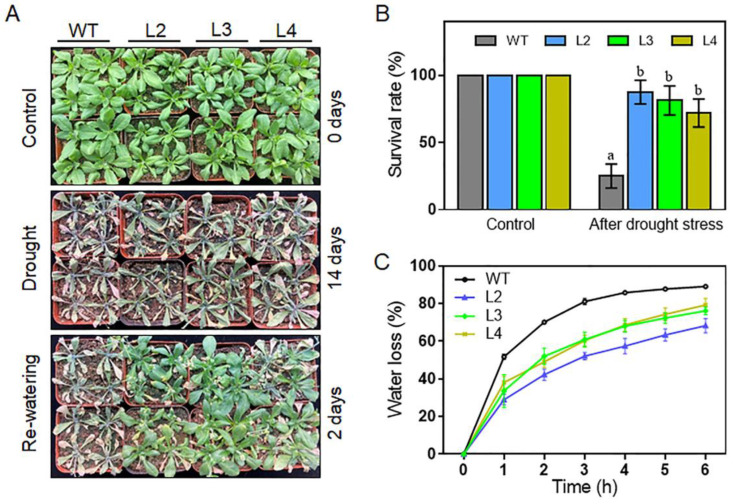
Comparison of drought tolerance among WT and *TaHsfA2e-5D* overexpressing transgenic lines. (**A**) Morphology and (**B**) survival rates of WT and transgenic lines (L2, L3 and L4) after drought treatment. The 21-day-old seedlings did not receive watering for 14 days until the plants withered. Afterwards, re-watering was applied, and survival rates were measured after re-watering for 2 days under normal conditions. Different lowercase letters above the bars denote significant differences at the *p* < 0.05 level. (**C**) Water loss rates of WT and transgenic lines. Values are means ± SD of three replicates.

Considering that heat stress and drought stress are closely correlated and that the expression of *TaHsfA2e-5D* is highly up-regulated by dehydration (PEG6000) stress (Figure 1C), the phenotypes of the transgenic plants under drought stress were investigated. As shown in Figure 7A, after withholding water for 14 days, the WT leaves were severely dehydrated, whereas the three transgenic lines exhibited better growth status than WT. After two days’ recovery, only 25% of the WT plants survived, whereas more than 70% of the transgenic plants survived in lines L2, L3, and L4 (Figure 7B). Additionally, the transgenic lines showed lower rates of water loss than the WT plants (Figure 7C). These data demonstrate that the *TaHsfA2e-5D* gene also confers drought tolerance in transgenic *Arabidopsis*.

### 2.7. Expression Analysis of Stress-Related Genes in TaHsfA2e-5D Transgenic Arabidopsis

**Figure 8:**
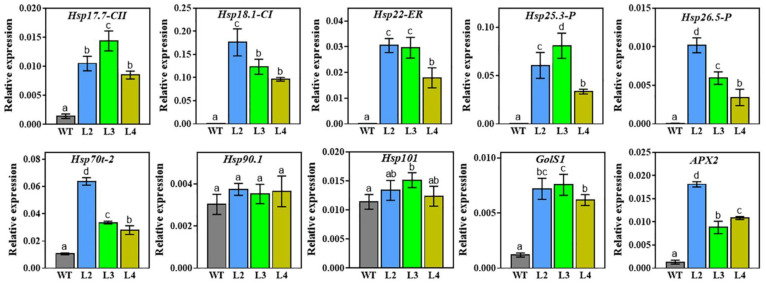
Expression of stress-related genes in WT and transgenic lines. Total RNAs were isolated from WT and transgenic plants grown for 10 days under normal conditions. Three biological replicates were used for each line. The *Arabidopsis Actin2* gene was used as an internal standard. All data represent means ± SD. Different lowercase letters above the bars denote significant differences at the *p* < 0.05 level. Hsp17.7-CII (AT5G12030), Hsp18.1-CI (At5g59720), Hsp22-ER (At4g10250), Hsp25.3-P (At4g27670), Hsp26.5-P (At1g52560), Hsp70t-2 (At2g32120), Hsp90.1 (At5g52640), Hsp101 (At1g74310), GolS1 (At2g47180), and APX2 (At3g09640).

To explore the molecular basis of heat and drought resistance conferred by *TaHsfA2e-5D* in transgenic *Arabidopsis*, the expression of several stress-responsive genes was examined in WT and transgenic lines by RT-qPCR. Specifically, ten genes positively regulated by *AtHsfA2* [26,27], including *Hsp17.7-CII*, *Hsp18.1-CI*, *Hsp22-ER*, *Hsp25.3-P*, *Hsp26.5-P*, *Hsp70t-2*, *Hsp90.1*, *Hsp101*, *GolS1*, and *APX2* were selected for analyses. As shown in Figure 8, the expression levels of most genes examined were significantly higher in *TaHsfA2e-5D* overexpressing lines than in WT, except that there was no significant difference between the WT and transgenic lines for *Hsp90.1* and *Hsp101* (Figure 8). These results revealed that the enhanced heat and drought tolerance conferred by *TaHsfA2e-5D* was mediated at least in part by the transcriptional activation of Hsp genes and other stress-related genes.

## 3. Discussion

High temperatures and drought are increasingly becoming a serious threat to wheat production worldwide. An increase in atmospheric temperature of 1 °C above the wheat’s optimal growing temperature during the reproductive development phase has been presumed to reduce wheat yield by 6–10%, which can greatly threaten food security [28]. It is known that transcription factors play important roles in mediating environmental stress responses by regulating the expression of stress-responsive genes [29]. Among them, Hsfs, which perform a pivotal role in plant adaptation to abiotic stresses, have been extensively studied in various plants [30]. Although 82 non-redundant Hsfs located on 21 chromosomes have been identified in wheat [12], fewer Hsfs have been cloned and characterized because of the complexity of the wheat genome. In this study, we cloned a wheat Hsf gene, *TaHsfA2e-5D*, which is located on chromosome 5D and the deduced protein features the DBD and OD motifs of HsfAs. Gene expression profiling revealed differential accumulation of *TaHsfA2e-5D* transcripts in all test tissues (Figure 1A), suggesting that it might play a role in plant growth and development. In spite of high sequence similarities between *TaHsfA2e-5D* and *TaHsfA2-1* (Figure 2C), the expression patterns of these two genes are quite different. *TaHsfA2-1* is specifically highly expressed in the mature leaf and lowly expressed in the young root and stamen [14]. Here, it was found that *TaHsfA2e-5D* transcripts were more abundant in the root and stamen, but less in the leaf (Figure 1A). *TaHsfA2-1* was upregulated by heat, drought, H_2_O_2_ and SA treatments [14]. In this study, *TaHsfA2e-5D* was also induced strongly by heat and drought, but weakly by H_2_O_2_ and SA treatments (Figure 1). These results suggest that although more than 82 Hsf genes have been found in the wheat genome, further study will be required to elucidate their individual roles.

The sequence of TaHsfA2e-5D is highly similar to its ortholog from diploid progenitor *Aegilops tauschii*, indicating that TaHsfA2e-5D is highly conserved during polyploidization. Homology analyses showed that TaHsfA2e-5D is homologous to *Arabidopsis* AtHsfA2 and rice OsHsfA2e (Figure 2C). *AtHsfA2* and *OsHsfA2e* have been reported to be able to respond to various environmental stresses [26,31]. In the present study, *TaHsfA2e-5D* was induced by various abiotic stresses and phytohormones (Figure 1), suggesting versatile functions of *TaHsfA2e-5D*, similar to its orthologous genes AtHsfA2 and OsHsfA2e. It was found that TaHsfA2e-5D contained the nuclear localization signal (NLS) sequence (Figure S1), and the subcellular localization assay in tobacco leaf epidermal cells confirmed that TaHsfA2e-5D is a nuclear localized protein (Figure 4A). Similar nuclear localization studies have been reported in AtHsfA2 and OsHsfA2e [26,31]. In addition, TaHsfA2e-5D shows a transcriptional activation activity, and a deletion analysis revealed that the activation domain of TaHsfA2e-5D is localized in the 300–348 region (Figure 4B), which contains an AHA motif (Figure S1) typical of HsfAs [10].

Like other Hsfs, *TaHsfA2e-5D* was found to confer significant thermotolerance on plants (Figure 5 and Figure 6). Besides, transgenic *Arabidopsis* lines clearly exhibited better growth status, higher survival rates, and lower water loss rates under drought stress conditions (Figure 7). In recent years, some heat shock factors have been reported to play important roles in other abiotic stresses in addition to heat stress. For example, *Arabidopsis* plants overexpressing *AtHsfA7b* exhibited enhanced tolerance against salt and drought stresses [32]. Maize *ZmHsf04* conferred increased thermo and salt-stress tolerance in transgenic *Arabidopsis* [33]. *ZmHsf05* improved thermotolerance in *Arabidopsis* and enhanced drought tolerance in rice [34,35], while overexpression of *ZmHsf08* reduced salt and drought tolerance in maize [30]. The recently reported *OsHSFA3* played an important role in ABA-mediated drought tolerance in *Arabidopsis* [36]. *TaHsfA6f* from wheat could enhance tolerance to heat, drought, and salt stresses of transgenic plants [16,17].

To adapt to heat and drought stresses, plants have developed different response strategies, including modulation of the expression of stress-related genes [37]. Hsps, as key molecular chaperones, assist with the folding, intracellular distribution, and degradation of proteins and thus play central roles in protecting plants from stress damage [6]. Proactive regulation of Hsfs to Hsps is particularly important in abiotic stress responses [37]. In this study, the expression levels of several Hsp genes, such as *Hsp17.7-CII*, *Hsp18.1-CI*, *Hsp22-ER*, *Hsp25.3-P*, *Hsp26.5-P*, and *Hsp70* were elevated significantly in *TaHsfA2e-5D* overexpressing *Arabidopsis* under normal conditions. However, no significant differences in the transcript levels of *Hsp90.1* and *Hsp101* genes were observed between transgenic and WT plants (Figure 8). This result is not consistent with those of previous studies on AtHsfA2 and OsHsfA2e, in which both genes were directly regulated by AtHsfA2 and OsHsfA2e [26,31]. Beyond Hsp genes, other stress-related genes, *APX2* and *GolS1*, were also up-regulated in *TaHsfA2e-5D* overexpression plants. The *APX2* gene encodes cytosolic ascorbate peroxidase which plays an important role in maintaining the activity of the antioxidant system and protects plants against oxidative damage resulting from adverse stresses [38]. The *GolS1* gene encodes galactinol synthase and was previously reported to function primarily in drought, high-salinity, and osmotic stress tolerance [39]. These results indicated that *TaHsfA2e-5D* positively regulates heat and drought stress responses, at least partly through the boosted expression of stress-responsive genes. Future studies will focus on the elucidation of the stress-responsive regulatory network underlying *TaHsfA2e-5D*, and the development of heat-/drought-resistance wheat cultivars through genetic manipulation, thereby improving wheat yield under environmental stress conditions.

In conclusion, we cloned and characterized a wheat Hsf gene, *TaHsfA2e-5D*, which was localized in the nucleus and was demonstrated to function as a transcriptional activator. Overexpression of *TaHsfA2e-5D* in yeast and *Arabidopsis* led to increased tolerance to heat and drought stresses. Furthermore, RT-qPCR analyses demonstrated that *TaHsfA2e-5D* functions through modulating the expression of Hsp genes and other stress-related genes. Taken together, our results suggest that *TaHsfA2e-5D* could be useful in molecular breeding of wheat or other crops for enhanced stress tolerance, especially during terminal heat and drought stresses.

## 4. Materials and Methods

### 4.1. Plant Materials, Growth Conditions, and Stress Treatments

Heat-/drought-resistant wheat cultivar TAM107 used for gene cloning and expression analysis was planted in Hoagland solution. Chinese Spring (CS) nulli-tetrasomic (NT) wheat lines (N5AT5B, N5AT5D, N5BT5A, N5BT5D, N5DT5A and N5DT5B) used for determining chromosomal locations, and tobacco (*Nicotiana benthamiana*) used for transient transfection were cultivated in soil. Columbia-0 (*Arabidopsis thaliana*) used for genetic transformation was sown on Murashige and Skoog (MS) agar media or in soil. All the plants were grown in a greenhouse at 22 °C with a 16-h photoperiod.

To explore the tissue-specific expression of *TaHsfA2e-5D*, we harvested the root, stem, leaf, young spike, glume, arista, stamen and pistil from TAM107 at the booting stage. To study the expression patterns of *TaHsfA2e-5D* in response to stresses, plants were grown hydroponically in a light chamber with a 16-h photoperiod at 22 °C, and 10-day-old wheat seedlings were treated by submerging the roots in Hoagland solution with 20% PEG 6000 (polyethylene glycol 6000), 0.2 M NaCl, 0.1 mM MeJA, 0.2 mM ABA, 10 mM H_2_O_2_, or 0.1 mM SA. For high-temperature and low-temperature stresses, the seedlings were transferred to another growth chamber with a constant temperature of 40 °C or 4 °C and with the same parameters of light and humidity as the previous chamber. For each treatment, five seedlings were separately sampled at 0, 1, 2, 4, 6, 12, and 24 h after treatment. All the samples were stored at −80 °C for RNA extraction.

### 4.2. Gene Cloning, Chromosomal Location, and Sequence Analysis

Genomic DNA and total RNA were isolated using the CTAB and TRIzol methods (Coolaber, Beijing, China), respectively. First-strand cDNA was synthesized using a Reverse Transcription System (Vazyme, Nanjing, China). Sequence BLAST search was performed through NCBI (https://www.ncbi.nlm.nih.gov/, accessed on 28 February 2022) and IWGSC (http://www.wheatgenome.org/, accessed on 28 February 2022) databases. The genomic and cDNA sequences of *TaHsfA2e-5D* were obtained from the DNA and cDNA of TAM107 by PCR with gene-specific primers. The chromosomal location of *TaHsfA2e-5D* was determined by PCR using chromosome specific primers with the DNA of CS NT lines as templates. All PCR reactions were performed using high-fidelity Tks Gflex™ DNA Polymerase (TaKaRa, Dalian, China) according to the manufacturer’s recommended procedures. The PCR products were constructed into the pEASY-Blunt cloning vector (TransGen, Beijing, China) for sequencing. Primer sequence information is presented in Table S1. DNAMAN8 software was used for sequence alignment. Cis-acting regulatory elements were predicted using PlantCARE (http://bioinformatics.psb.ugent.be/webtools/plantcare/html/, accessed on 28 February 2022). Phylogenetic tree was constructed using MEGA7 software, and sequence logos were analyzed by WebLogo (http://weblogo.berkeley.edu/, accessed on 28 February 2022) platform.

### 4.3. Gene Expression Analysis

Gene expression was analyzed by RT-qPCR using SYBR Green Realtime PCR Master Mix (Takara, Dalian, China) on a CFX96 real-time PCR machine (Bio-Rad, Hercules, CA, USA) following the manufacturer’s recommended procedures. The gene expression values were normalized to that of wheat *β-actin* or *Arabidopsis Actin2* using the 2^−∆∆CT^ method [40].

### 4.4. Subcellular Localization and Transactivation Analysis

The coding sequence (CDS) of *TaHsfA2e-5D* without a stop codon was inserted into the pCAM35s-GFP vector via an In-Fusion Cloning System (Vazyme, Nanjing, China). pCAM35s-GFP and pCAM35s-TaHsfA2e-5D-GFP were transformed respectively into tobacco leaf epidermal cells using the *Agrobacterium* infiltration method [41]. Fluorescence signals were visualized and photographed using a laser confocal microscope (Olympus, Tokyo, Japan).

The full-length and truncated *TaHsfA2e-5D* were respectively introduced into the pGBKT7 vector by the In-Fusion technique. The constructs, including negative control pGBKT7, positive control pGBKT7–P53, pGBKT7-TaHsfA2e-5D and pGBKT7-TaHsfA2e-5D C∆48 (without the C-terminal 48 amino acids) were respectively transformed into yeast strain AH109. Transcriptional activation activities were evaluated according to the growth status of transformants on SD/-Trp and SD/-Trp-His media.

### 4.5. Transformation and Thermotolerance Assay in Yeast

The CDS of *TaHsfA2e-5D* was cloned into yeast expression vector pYES2. The pYES2 contains a URA3 selection marker driven by a galactose-inducible (GAL1) promoter. The pYES2-TaHsfA2e-5D and the empty pYES2 control plasmids were transformed into yeast strain INVSc1, respectively. Positive transformants were screened and cultivated at 30 °C in an uracil-deficient synthetic complete (SC-ura) medium with 2% (*w*/*v*) glucose. A single positive clone of INVSc1 containing the fused pYES2-TaHsfA2e-5D or the empty pYES2 vector was inoculated in 5 mL of SC-ura liquid medium containing 2% glucose at 30 °C. For heat shock treatment, yeast cells which had been cultured overnight were diluted to an OD600 value of 0.4 into 10 mL induction SC-ura liquid medium (supplemented with 2% galactose). After incubation for approximately 16 h, the yeast cell densities were recalculated and an equal number of yeast cells were re-suspended in 200 μL of sterile water and exposed to normal conditions (30 °C) or heat stress conditions (40 °C or 50 °C) for 1 h. Afterwards, 20 μL of 10-fold serial dilutions were dotted on SC-ura plates, and incubated at 30 °C for 2 days to evaluate the growth status and relative survival rates. Meanwhile, the overnight cultures were resuspended and diluted in SC-ura liquid medium (supplemented with 2% galactose) to obtain an OD600 of 0.08. Furthermore, the dilutions were incubated at normal conditions (30 °C) or heat stress conditions (40 °C), and the OD600 was measured every 3 h to obtain the growth curves.

### 4.6. Arabidopsis Transformation and Stress Treatments

The CDS of *TaHsfA2e-5D* was cloned into pSuper1300 vector to generate a *TaHsfA2e-5D* overexpressing construct. The construct was transformed into wild-type (Columbia-0) *Arabidopsis* using the *Agrobacterium* mediated floral-dip method [42]. Single T-DNA insertion and homozygous transgenic lines were screened on MS media containing 25 mg L^−1^ hygromycin. The expression levels of transgenic plants were analyzed by RT-qPCR.

For heat stress treatment, sterilized seeds of WT and homozygous transgenic lines were planted on MS agar plates. Seven-day-old seedlings of WT and homozygous transgenic lines were placed at 45 °C for 2 h, and the survival rates were calculated after 3-day recovery at 22 °C. Additionally, 7-day-old seedlings were transplanted into nutrient-rich soil for 14 days under normal conditions. The 21-day-old *Arabidopsis* seedlings (the transgenic lines and the WT plants) were grown at 40/32 °C (day/night) with irrigation every day for 7 days. Then, the phenotypes and survival rates of plants were measured. For drought stress treatment, the 21-day-old *Arabidopsis* seedlings did not receive watering for 14 days until the plants withered. Afterwards, re-watering was applied, and survival rates were measured after re-watering for 2 days under normal conditions. Water loss rate assay was performed according to a previous study [43]. The above experiments were repeated three times.

### 4.7. Statistical Analyses

Fisher’s LSD multiple comparisons test following One-Way ANOVA was performed for WT and transgenic *Arabidopsis* lines overexpressing *TaHsfA2e-5D* by Prism 7 (GraphPad Software, Version 7.00, San Diego, CA, USA). Differences in relative survival rates between yeast harboring pYES2 and yeast with pYES2-TaHsfA2e-5D were evaluated using a Student’s *t* test.

## Data Availability

Not applicable.

## Supplementary Materials

The following supporting information can be downloaded at: https://www.mdpi.com/article/10.3390/ijms23052784/s1.
