# Supplementary material for: Characterization of the Wheat Heat Shock Factor TaHsfA2e-5D Conferring Heat and Drought Tolerance in Arabidopsis
Source: Int J Mol Sci. 2022 Mar 3;23(5):2784. doi: 10.3390/ijms23052784 (PMC8911409; doi:10.3390/ijms23052784)
Supplement: Supplementary file 1 [file ijms-23-02784-s001.zip › ijms-1593961-supplementary.pdf]

Supplementary Figures and Tables

```
1   ATGATGAATCCGGTCAAGGTGGAGGGCCGCCCCACCCCCACCCCCTCCCCGGCGGCCGCAGGCGGCGGAGGGGCGCCGAGGCCGATGGACGGGCTGGGCGACGCCGGGCCCACGCCGTTC
1    M  M  N  P  V  K  V  E  G  R  P  T  P  T  P  S  P  A  A  A  G  G  G  G  A  P  R  P  M  D  G  L  G  D  A  G  P  T  P  F

121 CTCGCCAAGACCTACGACATGGTCGACGACCCCGCCACCGACGCCGTCGTCTCCTGGACCGCCACCAGCAACAGCTTCGTCGTCTGGGACCCGCACCTCTTCGCCACCCTGCTGCTGCCG
41   L  A  K  T  Y  D  M  V  D  D  P  A  T  D  A  V  V  S  W  T  A  T  S  N  S  F  V  V  W  D  P  H  L  F  A  T  L  L  L  P

241 AGCTACTTCAAGCACAGCAACTTCTCCAGCTTCGTCCGACAGCTCAACACCTATGGCTTCAGAAAGGTGGATCCTGACAGGTGGGAATTTGCAAATGAGGGATTCTTGCGAGGGCAGAGG
81   S  Y  F  K  H  S  N  F  S  S  F  V  R  Q  L  N  T  Y  G  F  R  K  V  D  P  D  R  W  E  F  A  N  E  G  F  L  R  G  Q  R

361 CACCTTCTCAGAAATATTAAGCGCCGGAAACCTACACACGGGTCTCAGAATCAGCAATCTCTTGCCTCCTACCTTGAGGTGGGAAACTTTGGACATGACGTGGAGATAGATCATTTGAAA
121  H  L  L  R  N  I  K  R  R  K  P  T  H  G  S  Q  N  Q  Q  S  L  A  S  Y  L  E  V  G  N  F  G  H  D  V  E  I  D  H  L  K

481 AGGGACAAGCAGCTCTTGATGGCTGAAGTGGTCAAGCTCAGGCAGGAGCAACAGAACACAAGGTCAGGTCTGCAAGCCATGGAAAAGAGGCTGCAAGGAACCGAGCAGAAGCAACAGCAG
161  R  D  K  Q  L  L  M  A  E  V  V  K  L  R  Q  E  Q  Q  N  T  R  S  G  L  Q  A  M  E  K  R  L  Q  G  T  E  Q  K  Q  Q  Q

601 ATGATGTCGTTCTTGGCGCGAGTCATGCAGAATCCCGTTTTCATACGCCAGCTGATCTCCCAGAGTGAGATGAGGAAGGAGCTTGAAGATGCCATCTCAAATAAAAGACGGCGCCGCATC
201  M  M  S  F  L  A  R  V  M  Q  N  P  V  F  I  R  Q  L  I  S  Q  S  E  M  R  K  E  L  E  D  A  I  S  N  K  R  R  R  R  I

721 GACCAGGGACCTGAAGCCGTCGATAGCATGGGCACTGGCTCTACCCTGGAGCAAGGGTCACATGTAATGTTTGAGCAGCAGGAGCCAGTGGATTCATTCGTGAACGGTGTCATATCCGAT
241  D  Q  G  P  E  A  V  D  S  M  G  T  G  S  T  L  E  Q  G  S  H  V  M  F  E  Q  Q  E  P  V  D  S  F  V  N  G  V  I  S  D

841 CTTGAAAGCTCGTCCGTCGATACAAAGGGAGCTGAGGTGCAGCAGAGTGTTGCTTCCAGCCGTTCGGAGCAACTGAGAGGCAGGCCCAGTGGAGAGCTAAATGATGATTTCTGGGAGGAC
281  L  E  S  S  S  V  D  T  K  G  A  E  V  Q  Q  S  V  A  S  S  R  S  E  Q  L  R  G  R  P  S  G  E  L  N  D  D  F  W  E  D

961 CTGCTGCATGAAGGGGGGCTCGGCGAGGAGGCCAGCAACCTTGTGGTTCCAGATGACATGAACTTGTTGGCTGAGAAGCTGGACTAG
321  L  L  H  E  G  G  L  G  E  E  A  S  N  L  V  V  P  D  D  M  N  L  L  A  E  K  L  D  *
```

Figure S1. Nucleotide and corresponding amino acid sequences of *TaHsfA2e-5D*. The important domains of TaHsfA2e-5D are indicated as follows: DBD (red), OD (blue), NLS (orange), NES (black underline) and AHA (green).

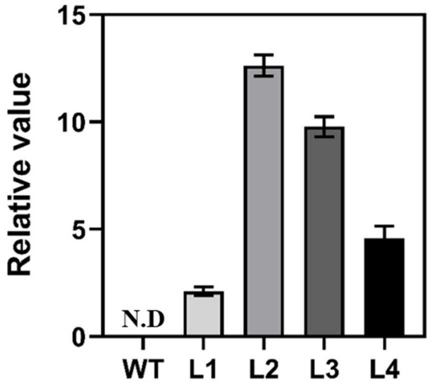

Figure S2. Transcript levels of TaHsfA2e-5D in 7-day-old WT and transgenic lines. N.D, Not Detected. *Arabidopsis Actin2* gene was used as the internal standard. Error bars represent means ± SD.

Table S1. Primer sequences used in this study.

Name	Sequence (5´-3´)	Application
TaHsfA2e-5D-GF	TATCTCTCGTGCCTCCCTG	Genomic amplification Chromosomal location
TaHsfA2e-5D-GR	TGCGATGGATTATTGTCTGG	
TaHsfA2e-5D-CF	TATCTCTCGTGCCTCCCTG	cDNA amplification
TaHsfA2e-5D-CR	TGCGATGGATTATTGTCTGG	
TaHsfA2e-5D-PF	TGGACCAACGAATCACTG	Promoter amplification
TaHsfA2e-5D-PR	CAGGGAGGCACGAGAGATA	
TaHsfA2e-5D-F	CAGGAGCCAGTGGATTCA	RT-qPCR analysis
TaHsfA2e-5D-R	GGAATTCCTGTTCTTCGAGTAG	
β-Actin-F	GACCGTATGAGCAAGGAGAT	
β-Actin-R	CAATCGCTGGACCTGACTC	
Hsp17.7-CII-F	TCAGGTCCAGATAGAGAACGAGAAC	
Hsp17.7-CII-R	CCTCTCCATCCTCACAAACTTCAC	
Hsp18.1-CI-F	GGTTACCGGAGAATGCAAAGATG	
Hsp18.1-CI-R	CGATGGACTTGACTTGAGGCTTC	
Hsp22-ER-F	GCTTGAGAATGGTGTGCTCAC	
Hsp22-ER-R	CATGACACTCCATTTAGCTTACAC	
Hsp25.3-P-F	ACGGAGTCCTCTTTATCACTATCCC	
Hsp25.3-P-R	GATCGAGTCCTACTGAATCTGG	
Hsp26.5-P-F	ACCTGAGGAAGACGAGTACTGGTC	
Hsp26.5-P-R	GCCTTAATGTCCTCAACCTTAGC	
Hsp70t-2-F	CTGTTAACCATACAAGCCACACCTC	
Hsp70t-2-R	TGTGTTACGCGGAATCACG	
Hsp90.1-F	AGATGGCGGTGTCTTGTCACAG	
Hsp90.1-R	CCATCGCAACGAACTTTG	
Hsp101-F	GCCTTACTTCTGCCAAATGCG	
Hsp101-R	AGATGGCGGTGTCTTGTCACAG	
GolS1-F	AAACCGCTGATGCTATGTCC	
GolS1-R	TCACGTAATCACCGTTTCCA	
APX2-F	AAGTTGAGCCACCTCCTGAA	
APX2-R	GTGTGTCCACCAGACAATGC	
Actin2-F	GCTCCTCTTAACCCAAAGGC	
Actin2-R	CACACCATCACCAGAATCCAGC	
TaHsfA2e-5D-SF	GGGGACGAGCTCGGTACCATGATGAATCCGGTC	Subcellular localization
TaHsfA2e-5D-SR	CATGGTGTCGACTCTAGAGTCCAGCTTCTCAGC	
TaHsfA2e-5D-AF	TGGCCATGGAGGCCGAATTCATGATGAATCCGGTC	Transactivation analysis
TaHsfA2e-5D-AR	CGACGGATCCCCGGGAATTCGTCCAGCTTCTCAGC	
TaHsfA2e-5D-TYF	CCGCCAGTGTGCTGGAATTCATGATGAATCCGGTC	Transgenic yeast
TaHsfA2e-5D-TYR	GATGGATATCTGCAGAATTCGTCCAGCTTCTCAGC	
TaHsfA2e-5D-TAF	TACACCAAATCGACTCTAGAATGATGAATCCGGTC	Transgenic *Arabidopsis*
TaHsfA2e-5D-TAR	ATAGGTACCCGGGCTCTAGAGTCCAGCTTCTCAGC	
